# Urticaria in Monozygotic and Dizygotic Twins

**DOI:** 10.1155/2012/125367

**Published:** 2012-11-20

**Authors:** Simon Francis Thomsen, Sophie van der Sluis, Kirsten Ohm Kyvik, Vibeke Backer

**Affiliations:** ^1^Department of Dermato-Allergology Gentofte Hospital 2900 Hellerup, Denmark; ^2^Complex Trait Genetics, Department of Functional Genomics and Department of Clinical Genetics, Center for Neurogenomics and Cognitive Research, VU University Medical Center, Neuroscience Campus Amsterdam, 1007 MB Amsterdam, The Netherlands; ^3^Institute of Regional Health Services Research and Odense Patient Data Explorative Network, University of Southern Denmark, 5000 Odense, Denmark; ^4^The Danish Twin Registry, University of Southern Denmark, 5000 Odense, Denmark; ^5^Department of Respiratory Medicine, Bispebjerg Hospital, 2400 Copenhagen NV, Denmark

## Abstract

*Aim*. To identify risk factors for urticaria, to determine the relative proportion of the susceptibility to urticaria that is due to genetic factors in an adult clinical twin sample, and to further determine whether the genetic susceptibility to urticaria overlaps with the genetic susceptibility to atopic diseases. *Methods*. A total of 256 complete twin pairs and 63 single twins, who were selected from sibships with self-reported asthma via a questionnaire survey of 21,162 adult twins from the Danish Twin Registry, were clinically interviewed about a history of urticaria and examined for atopic diseases. Data were analysed with Cox proportional hazards regression and variance components models. *Results*. A total of 151 individuals (26%) had a history of urticaria, whereas 24 (4%) had had symptoms within the past year. Female sex, HR = 2.09 (1.46–2.99), *P* = 0.000; hay fever, HR = 1.92 (1.36–2.72), *P* = 0.000; and atopic dermatitis, HR = 1.44 (1.02–2.06), *P* = 0.041 were significant risk factors for urticaria. After adjustment for sex and age at onset of urticaria in the index twin, the risk of urticaria was increased in MZ cotwins relative to DZ cotwins, HR = 1.42 (0.63–3.18), *P* = 0.394. Genetic factors explained 45% (16–74%), *P* = 0.005, of the variation in susceptibility to urticaria. The genetic correlation between urticaria and hay fever was 0.45 (0.01–0.89), *P* = 0.040. *Conclusions*. Susceptibility to urticaria is partly determined by genetic factors. Urticaria is more common in women, and in subjects with hay fever and atopic dermatitis, and shares genetic variance with hay fever.

## 1. Introduction

Urticaria is a common complex disease characterised by disseminated or local eruption of migrating itching wheals sometimes complicated by swelling of mucosal membranes; angioedema. Urticaria carries a lifetime risk of about 20% and is a troublesome disease that significantly impacts on quality of life. The mainstay of treatment of urticaria is nonsedating antihistamines. However, in many patients treatment with immunosuppressive drugs such as corticosteroids, azathioprine, cyclosporine, and biological therapies is necessary in order to obtain symptomatic control [1, 2].

 Urticaria can be divided into acute and chronic types arbitrarily based on whether symptoms have been present for more than six weeks. Acute urticaria is usually triggered by allergic reactions to foods, insect venoms, or drugs, or it can be triggered by infections. However, in many cases the cause remains unknown. Likewise, chronic urticaria can be classified into several clinical and aetiological subtypes of for example allergic, autoimmune, physical, or idiopathic origin. Particularly, chronic urticaria that has no detectable cause is termed chronic idiopathic urticaria, whereas chronic autoimmune urticaria is present in those with antibodies against the high-affinity IgE receptor (Fc*ε*RI) on mast cells and basophils [3]. 

Genetic studies of urticaria have shown very diverse results, in part due to the heterogeneous nature of the disease. Notably, polymorphisms in multiple genes such as the TNF-alpha promoter, the IL18, the thromboxane A2 receptor, the leukotriene C4 synthase, and the Fc*ε*RI genes, have been associated with aspirin-sensitive urticaria [4–9]. Moreover, studies have associated several variants in the HLA, the FPRL1 promoter, and the TGF-beta1 promoter and cyclo-oxygenase and 5-lipo-oxygenase-activating protein genes, with chronic urticaria [10–14]. Finally, hereditary angioedema has an obvious genetic cause being transmitted autosomal dominantly (disease types I and II) via mutations in the C1NH gene; disease type III is more heterogeneous and is caused by mutations in the Factor XII gene [15]. Taken together, these findings suggest a genetic component in the pathogenesis of several urticaria subtypes. However, to what extent genetic factors account for variation in overall urticaria susceptibility is less well-determined. One previous twin study of 1480 Swedish children, 7–9 years of age, found a heritability of urticaria of 54% in boys and 60% in girls [16]. However, the urticaria diagnosis relied on questionnaire responses and was not confirmed by clinical examination. We identified risk factors for urticaria and determined the relative proportion of the susceptibility to urticaria that was due to genetic factors in an adult clinical twin sample selected from sibships with self-reported asthma. Furthermore, we studied whether the genetic susceptibility to urticaria overlaps with the genetic susceptibility to atopic diseases.

## 2. Methods

### 2.1. Study Population

The sample consisted of twins born between 1953 and 1982, who were registered in the nationwide Danish Twin Registry [17]. A total of 21,162 twin individuals, 20–49 years of age, answered a questionnaire concerning health and lifestyle. The response rate was 75%. Of these, 7,317 twin pairs were intact and 6,528 subjects were single responders. In total 6,791 pairs had known zygosity (33% monozygotic (MZ) twins, and 67% dizygotic (DZ) twins) and complete data on asthma. Among these, 438 pairs who lived in the geographical region surrounding the study site, and of whom at least one proband had self-reported asthma based on an affirmative response to a screening question, were invited to take part in a clinical examination. The examination included an interview and paraclinical tests for allergy (measurement of serum total IgE and skin prick test for common aeroallergens). The asthma-screening question was “Do you have, or have you ever had asthma?” Twin zygosity was determined using four questions of similarity between the twins, which assign zygosity correctly in more than 95% of the cases [18]. The Scientific Ethics Committee of Copenhagen approved the study.

### 2.2. Clinical Interview

At the clinical interview, subjects were asked about a lifetime history of urticaria, age at first episode, presence of symptoms within the past year, and current symptoms. Specific questions were “Have you ever had nettle rash/hives/urticaria?,” “Have you had nettle rash/hives/urticaria within the past year?,” “How old were you when you got nettle rash/hives/urticaria?” The interviewing physician explained symptoms of nettle rash/hives/urticaria and made sure that each patient understood the term and provided as qualified an answer as possible. In cases of doubt, photographic material was used as diagnostic aid. By means of similar questions subjects were also asked about the presence of asthma [19], hay fever [20], and atopic dermatitis [21].

### 2.3. Skin Prick Test and Measurement of Serum Total IgE

Skin prick tests were performed using standard dilutions birch, grass, mugwort, horse, dog, cat, house dust mite (*Dermatophagoides pteronyssinus *and *Dermatophagoides farinae*), and mould (*Alternaria iridis* and *Cladosporium herbarium*) allergens (Soluprick SQ system; ALK-Abelló, Denmark). Reactions were read after 15 minutes. A positive result was defined as a positive reaction to at least one of the allergens, and a reaction was considered positive if the mean wheal diameter was at least 3 mm. Serum total IgE was measured with an enzyme-linked immunosorbent assay (ELISA) on Immulite 2500 (DPC, New York, USA). Results were expressed as KIU/L. 

### 2.4. Statistical Analysis

First, risk factors for urticaria were determined with a Cox proportional hazards regression model with age as the underlying time ignoring the familial relationships between the twins. Covariates were sex, BMI, zygosity, asthma, hay fever, atopic dermatitis, smoking ever, positive skin prick test, and serum total IgE. Next, a Cox proportional hazards regression model was fitted with the time to onset of urticaria in the cotwin of an affected twin (the index twin) as the underlying time, and zygosity, sex, and age at onset of urticaria in the index twin as covariates. In this analysis an increased hazard ratio (HR) in MZ twins relative to DZ twins would signal a genetic susceptibility to urticaria. Regression analyses were performed in SPSS 16.0 (SPSS, Inc., Chicago, IL, USA).

 Using the classical twin method [22], the variation in the susceptibility to urticaria was partitioned into components representing latent genetic and environmental factors with sex as covariate. Subsequently, bivariate variance components analyses were performed between urticaria and atopic diseases. These bivariate analyses allow one to test to what extent the correlation between diseases is of genetic or environmental origin [23]. Variance components analyses were performed in the statistical package SOLAR, in which estimates are adjusted for ascertainment due to the selection in this study through a twin proband with asthma.

## 3. Results

A total of 575 subjects (256 intact pairs; 89 MZ pairs, 167 DZ pairs, and 63 single twins) underwent the clinical examination (individual response rate 67%; pair-wise response rate 60%). Characteristics of the studied subjects are given in Table 1. The mean age was 36 years, 58% of the subjects were females, and 46% had a history of smoking. 

**Figure 1:**
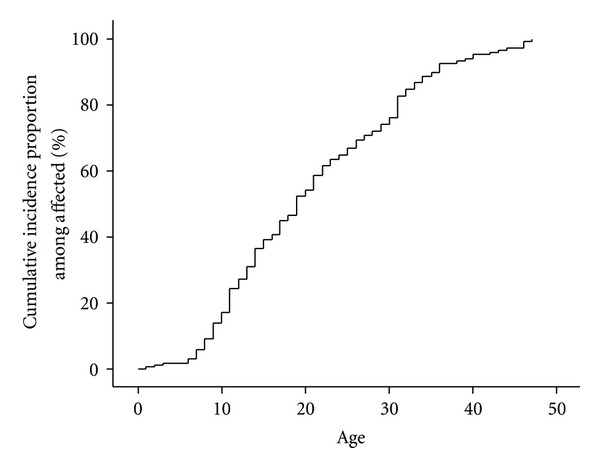
Cumulative incidence proportion of urticaria among all affected patients.

A total of 151 (26.3%) of the population; 43 (17.9%) of the men and 108 (32.2%) of the women had a history of urticaria, whereas 24 (4.2%); 6 (2.5%) of the men and 18 (5.4%) of the women had had symptoms within the past year. None of the subjects had urticaria at the day of examination. Among subjects with a history of urticaria, the median age at onset was 18.6 years (Figure 1). After proportional hazards regression, female sex, HR = 2.09 (1.46–2.99), *P* = 0.000; hay fever, HR = 1.92 (1.36–2.72), *P* = 0.000; and atopic dermatitis, HR = 1.44 (1.02–2.06), *P* = 0.041 were retained as significant risk factors for urticaria (Table 2).

**Figure 2:**
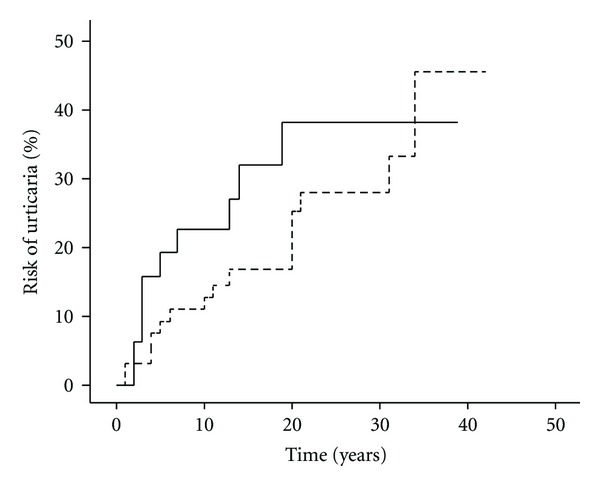
Risk of urticaria in the cotwin of an affected twin. Note: Full line represents monozygotic twins; dotted line represents dizygotic twins. Log rank *P* = 0.260.

Figure 2 shows the risk of urticaria in the cotwin of an affected twin for MZ and DZ twins separately, as a function of age of onset in the index twin. After adjustment for sex and age at onset of urticaria in the index twin, the risk of urticaria was increased in MZ cotwins relative to DZ cotwins, HR = 1.42 (0.63–3.18), *P* = 0.394, indicating that the variation in the susceptibility to urticaria is of genetic origin (Table 3). As this regression analyses only built on 34 MZ and 73 DZ twin pairs of whom 10 and 16, respectively, were concordant for urticaria, we proceeded with variance components analysis, which was based on the data of all twins in the sample.

 Variance components analysis showed that genetic factors explained 45% (16–74%), *P* = 0.005 of the variation in susceptibility to urticaria, 64% (33–95%), *P* = 0.000 of atopic dermatitis, 40% (24–56%), *P* = 0.000 of hay fever, and 25% (13–37%), *P* = 0.000 of asthma. The genetic and environmental correlations, respectively, between urticaria and atopic dermatitis were −0.06 (−0.58–0.46), *P* = ns and 0.23 (−0.32–0.78), *P* = ns; between urticaria and hay fever were 0.45 (0.01–0.89), *P* = 0.040 and −0.15 (−0.16–0.47), *P* = ns; and between urticaria and asthma were −0.07 (−0.62–0.49), *P* = ns and −0.22 (−0.78–0.34), *P* = ns, indicating that urticaria shares some genetic variance with hay fever, but not with atopic dermatitis and asthma.

## 4. Discussion

This study provides one among just a few available estimates of the heritability of urticaria and to our knowledge is the only clinical investigation of the heritability of the disease; genetic factors were found to explain about half of the variation in the susceptibility to urticaria, whereas the other half could be ascribed to random nongenetic variation. The lifetime prevalence of urticaria in the population was 26%, whereas the 12-month prevalence was 4%. The disease was about two times more common in women than in men and in subjects with hay fever and atopic dermatitis. Furthermore, urticaria was found to share genetic variance with hay fever, but not with atopic dermatitis or asthma. 

 One of the clear strengths of this study is the systematic selection of subjects from a nationwide population of twins, and the subsequent collection of detailed clinical interview data on 575 adults. Urticarial rash is transient and is therefore often not present at the day of examination. Therefore, the diagnosis relies chiefly on patient recollection and on the capability of the interviewing physician to obtain the correct information about past symptoms. Although we collected data on the lifetime history of urticaria, we did not obtain information on the duration and specific exposures relating to the occurrence of symptoms, which limits our ability to distinguish between different subtypes of disease. Particularly, we had no information on food allergy, allergy to medications, or other factors that could have aided in the aetiological workup. Also, we did not record whether urticaria episodes were accompanied by allergic reactions such as angioedema, acute gastroenteritis, airway symptoms, or anaphylaxis. Neither did we record whether the subjects had had several or just one episode of the disease. Common physical urticarias such as symptomatic dermographism, delayed pressure urticaria and cholinergic urticaria, and chronic spontaneous urticaria are entirely different dermatoses which all manifest as clinically different types of hives. The nature and importance of genetic factors will predictably differ in these diverse subtypes. Unfortunately the clinical interview did not allow discrimination between these different forms of chronic urticaria. However, a large proportion of the studied subjects had known type 1 allergy and associated atopic disorders, which makes it likely that their episodes of urticaria had an allergic basis. We therefore consider the main type of urticaria encountered in our population acute type 1 allergy-mediated urticaria and not chronic urticaria. This was also supported by our finding that the 12-month and current prevalence of the disease was much lower than the cumulative lifetime prevalence, and that the cumulative lifetime prevalence in this population with atopic predisposition was slightly higher than what could be expected in the general population. Our results may therefore only be applicable to an allergic population and not to the population as a whole, although we used statistical methods to correct for this skewed ascertainment of individuals. 

 Urticaria and hay fever were genetically correlated to a sizable extent; 0.45. Interestingly, in congruence with our finding, the genetic correlation between urticaria and hay fever was also exactly 0.45 among Swedish children [16]. In contrast, however, we did not find a significant genetic correlation between urticaria and atopic dermatitis or asthma, which is contradictory to the findings among Swedish children in whom the genetic correlation between urticaria and atopic dermatitis was 0.48, and the genetic correlation between urticaria and asthma was 0.48. 

However, the Swedish study was based on questionnaire responses while our study was based on confirmed clinical examination. A recent finding among a Han Chinese population showed that polymorphisms in the high affinity receptor of IgE are associated with atopic dermatitis but not with urticaria, indicating that these diseases may have different genetic aetiologies, at least in respect to certain genetic polymorphisms [24].

 We conclude that the susceptibility to urticaria is determined partly by genetic factors. Notably, half of all variance of urticaria susceptibility is ascribable to differences between subjects on a genetic level. Moreover, urticaria is more common in women and in subjects with hay fever and atopic dermatitis and has overlapping genetic features with hay fever. Future genetic studies distinguishing different subphenotypes of urticaria (e.g., chronic autoimmune or chronic idiopathic urticaria) could contribute to further unravelling of the aetiology of the disease. 

## Figures and Tables

**Table 1:** Descriptive characteristics of a sample of 575 Danish twins, 20–51 years of age.

	*N* (%)
Demographic data	
Age	35.9 (8.2)
Males	240 (41.7)
Females	335 (58.3)
MZ twins	191 (33.2)
DZ twins	384 (66.8)
BMI	25.3 (4.6)
Smoking ever	262 (45.6)
Clinical traits	
Urticaria ever	151 (26.3)
Urticaria in the past 12 months	24 (4.2)
Urticaria currently	0 (0)
Asthma	337 (58.6)
Hay fever	309 (53.7)
Atopic dermatitis	137 (23.8)
Paraclinical traits	
Positive skin prick test	318 (55.3)
Serum total IgE	54.4 (4.3)

MZ: monozygotic; DZ: dizygotic; BMI: body mass index.

All numbers are prevalence (%) except age (years, mean); BMI (kg/m^2^, mean); and serum total IgE (KIU/L, geometric mean).

**Table 2:** Predictors of urticaria in a sample of 575 Danish twins, 20–51 years of age.

	Urticaria difference (%)	Crude HR (95% CI)	*P* value	Adjusted HR (95% CI)	*P* value
Sex	14.3	1.96 (1.39–2.80)	0.000	2.09 (1.46–2.99)	0.000
BMI	0.2	0.98 (0.94–1.02)	0.249	ns	
Zygosity	1.7	1.01 (0.72–1.42)	0.963	ns	
Smoking ever	4.1	0.81 (0.58–1.11)	0.192	ns	
Asthma	9.7	1.60 (1.14–2.25)	0.007	ns	
Hay fever	13.9	1.93 (1.38–2.71)	0.000	1.92 (1.36–2.72)	0.000
Atopic dermatitis	10.6	1.63 (1.15–2.30)	0.006	1.44 (1.02–2.06)	0.041
Positive skin prick test	7.4	1.45 (1.04–2.01)	0.028	ns	
Serum total IgE	19.2	1.31 (1.03–1.68)	0.031	ns	

Urticaria difference is the absolute difference in prevalence between exposure groups; for example for sex the difference in the prevalence of urticaria between males and females is 14.3%. All numbers are (%) except BMI (difference in mean BMI between urticaria patients and non-urticaria patients) and serum total IgE (difference in mean serum total IgE (log-transformed) between urticaria patients and non-urticaria patients).

HR: hazard ratio; CI: confidence interval; BMI: body mass index. Adjusted HR is multivariably adjusted.

**Table 3:** Predictors of urticaria in the co-twin of an affected twin, in twin pairs, 20–51 years of age.

	Crude HR	*P* value	Adjusted HR	*P* value
	(95% CI)		(95% CI)	
Zygosity				
DZ	1.00		1.00	
MZ	1.57 (0.71–3.47)	0.267	1.42 (0.63–3.18)	0.394
Sex				
Males	1.00		1.00	
Females	1.86 (0.78–4.44)	0.160	1.86 (0.78–4.45)	0.165
Age at onset	0.98 (0.93–1.03)	0.328	0.98 (0.93–1.03)	0.346

DZ: dizygotic twins; MZ: monozygotic twins. HR: hazard ratio; CI: confidence interval. Adjusted HR is multivariably adjusted.

## References

[1] Zuberbier T, Asero R, Bindslev-Jensen C (2009). EAACI/GA2LEN/EDF/WAO guideline: definition, classification and diagnosis of urticaria. *Allergy*.

[2] Zuberbier T, Asero R, Bindslev-Jensen C (2009). EAACI/GA(2)LEN/EDF/WAO guideline: management of urticaria. *Allergy*.

[3] Brunetti L, Francavilla R, Miniello VL (2004). High prevalence of autoimmune urticaria in children with chronic urticaria. *Journal of Allergy and Clinical Immunology*.

[4] Kim SH, Son JK, Yang EM, Kim JE, Park HS (2011). A functional promoter polymorphism of the human IL18 gene is associated with aspirin-induced urticaria. *British Journal of Dermatology*.

[5] Choi JH, Kim SH, Cho BY (2009). Association of TNF-*α* promoter polymorphisms with aspirin-induced urticaria. *Journal of Clinical Pharmacy and Therapeutics*.

[6] Palikhe NS, Kim SH, Lee HY, Kim JH, Ye YM, Park HS (2011). Association of thromboxane A2 receptor (TBXA2R) gene polymorphism in patients with aspirin-intolerant acute urticaria. *Clinical and Experimental Allergy*.

[7] Bae JS, Kim SH, Ye YM (2007). Significant association of Fc*ε*RI*α* promoter polymorphisms with aspirin-intolerant chronic urticaria. *Journal of Allergy and Clinical Immunology*.

[8] Sánchez-Borges M, Acevedo N, Vergara C (2009). The A-444C polymorphism in the leukotriene C4 synthase gene is associated with aspirin-induced urticaria. *Journal of Investigational Allergology and Clinical Immunology*.

[9] Choi JH, Kim SH, Suh CH, Nahm DH, Park HS (2005). Polymorphisms of high-affinity IgE receptor and histamine-related genes in patients with ASA-induced urticaria/angioedema. *Journal of Korean Medical Science*.

[10] Yang EM, Kim SH, Kim NH, Park HS (2010). The genetic association of the FPRL1 promoter polymorphism with chronic urticaria in a Korean population. *Annals of Allergy, Asthma and Immunology*.

[11] Bozek A, Krajewska J, Filipowska B (2010). HLA status in patients with chronic spontaneous urticaria. *International Archives of Allergy and Immunology*.

[12] di Lorenzo G, Pacor ML, Candore G (2011). Polymorphisms of cyclo-oxygenases and 5-lipo-oxygenase-activating protein are associated with chronic spontaneous urticaria and urinary leukotriene E4. *European Journal of Dermatology*.

[13] Çoban M, Erdem T, Özdemir Ş, Pirim I, Atasoy M, Ikbal M (2008). HLA class I and class II genotyping in patients with chronic urticaria. *International Archives of Allergy and Immunology*.

[14] Hosseini-Farahabadi S, Tavakkol-Afshari J, Ganjali R, Rafatpanah H, Ghaffari J, Farid-Hosseini R (2006). Association between the polymorphism of TGF-*β*1 gene promoter (-509C>T) and idiopathic chronic urticaria. *Iranian Journal of Allergy, Asthma and Immunology*.

[15] Caballero T, Baeza ML, Cabañas R (2011). Consensus statement on the diagnosis, management, and treatment of angioedema mediated by bradykinin. Part I. Classification, epidemiology, pathophysiology, genetics, clinical symptoms, and diagnosis. *Journal of Investigational Allergology and Clinical Immunology*.

[16] Lichtenstein P, Svartengren M (1997). Genes, environments, and sex: factors of importance in atopic diseases in 7-9-year-old Swedish twins. *Allergy*.

[17] Skytthe A, Ohm Kyvik K, Vilstrup Holm N, Christensen K (2011). The Danish twin registry. *Scandinavian Journal of Public Health*.

[18] Christiansen L, Frederiksen H, Schousboe K (2003). Age- and sex-differences in the validity of questionnaire-based zygosity in twins. *Twin Research*.

[19] http://www.ginasthma.org/.

[20] Bousquet J, Van Cauwenberge P, Khaltaev N (2001). Allergic rhinitis and its impact on asthma. *Journal of Allergy and Clinical Immunology*.

[21] Hanifin JM, Cooper KD, Ho VC (2004). Guidelines of care for atopic dermatitis. *Journal of the American Academy of Dermatology*.

[22] Neale MC, Cardon LR (1992). *Methodology for Genetic Studies of Twins and Families*.

[23] Posthuma D, Beem AL, De Geus EJC (2003). Theory and practice in quantitative genetics. *Twin Research*.

[24] Zhou J, Zhou Y, Lin LH (2012). Association of polymorphisms in the promoter region of FCER1A gene with atopic dermatitis, chronic uticaria, asthma, and serum immunoglobulin E levels in a Han Chinese population. *Human Immunology*.

